# Green Adeptness in the Synthesis and Stabilization of Copper Nanoparticles: Catalytic, Antibacterial, Cytotoxicity, and Antioxidant Activities

**DOI:** 10.1186/s11671-017-2399-8

**Published:** 2017-12-28

**Authors:** Muhammad Imran Din, Farhan Arshad, Zaib Hussain, Maria Mukhtar

**Affiliations:** 1 Institute of Chemistry, University of Punjab, Lahore, 54590 Pakistan; 2 Department of Zoology, University of Punjab, Lahore, 54590 Pakistan

**Keywords:** Phytosynthesis, Copper nanoparticles, Phytochemicals, Cytotoxicity, Catalytic activity, Antibacterial activity

## Abstract

Copper nanoparticles (CuNPs) are of great interest due to their extraordinary properties such as high surface-to-volume ratio, high yield strength, ductility, hardness, flexibility, and rigidity. CuNPs show catalytic, antibacterial, antioxidant, and antifungal activities along with cytotoxicity and anticancer properties in many different applications. Many physical and chemical methods have been used to synthesize nanoparticles including laser ablation, microwave-assisted process, sol-gel, co-precipitation, pulsed wire discharge, vacuum vapor deposition, high-energy irradiation, lithography, mechanical milling, photochemical reduction, electrochemistry, electrospray synthesis, hydrothermal reaction, microemulsion, and chemical reduction. Phytosynthesis of nanoparticles has been suggested as a valuable alternative to physical and chemical methods due to low cytotoxicity, economic prospects, environment-friendly, enhanced biocompatibility, and high antioxidant and antimicrobial activities. The review explains characterization techniques, their main role, limitations, and sensitivity used in the preparation of CuNPs. An overview of techniques used in the synthesis of CuNPs, synthesis procedure, reaction parameters which affect the properties of synthesized CuNPs, and a screening analysis which is used to identify phytochemicals in different plants is presented from the recent published literature which has been reviewed and summarized. Hypothetical mechanisms of reduction of the copper ion by quercetin, stabilization of copper nanoparticles by santin, antimicrobial activity, and reduction of 4-nitrophenol with diagrammatic illustrations are given. The main purpose of this review was to summarize the data of plants used for the synthesis of CuNPs and open a new pathway for researchers to investigate those plants which have not been used in the past.

**Graphical abstract:**
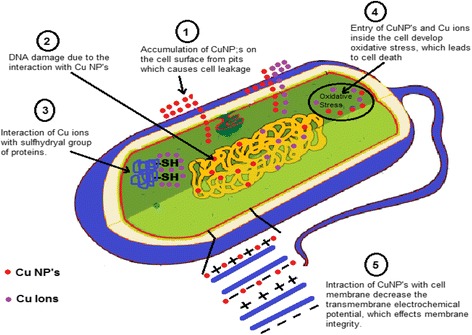
Proposed Mechanism for Antibacterial activity of copper nanoparticles.

Proposed Mechanism for Antibacterial activity of copper nanoparticles.

## Background

Nanoparticles (NPs) have a number of interesting applications in the industrial field such as space technology, magnetism, optoelectronics and electronics, cosmetics, and catalytic, pharmaceutical, biomedical, environmental, and energy applications [1, 2]. The extraordinary properties of NPs such as ductility, high yield strength, hardness, flexibility, rigidity, high surface-to-volume ratio, macroquantum tunneling effect, and quantum size are attributable as compared to properties of bulk materials having the same chemical composition [3]. Indeed, the properties of NPs, which may considerably differ from those observed for fine particles, are higher specific surface area, specific optical properties, lower melting points, specific magnetizations, mechanical strength, and numerous industrial applications [4]. Copper nanoparticles (CuNPs) are of great interest due to easy availability, low cost, and their similar properties to those of noble metals [5–9]. CuNPs can also be used in sensors, heat transfer systems [10–12], and electronics (fuel cell and solar cell), as catalysts in many reactions and as bactericidal and antimicrobial agents used to coat hospital equipment [13–19].

Many physical and chemical methods including laser ablation [20], microwave-assisted process, sol-gel [21], co-precipitation [22], pulsed wire discharge [23], vacuum vapor deposition [24], high-energy irradiation [25], lithography [26], mechanical milling [27], photochemical reduction, electrochemistry [28–32], electrospray synthesis [33], hydrothermal reaction [34], microemulsion [35], and chemical reduction are used to synthesize nanoparticles. Although physical and chemical methods produce well-defined and pure nanoparticles, these methods are neither cost-effective nor eco-friendly due to the use of toxic chemicals. One of the most important criteria of nanotechnology is the development of eco-friendly, nontoxic, and clean green chemistry procedures [36]. Hence, biosynthesis of nanoparticles contains a green chemistry-based method which employs different biological bodies such as plants [37, 38], actinomycetes [39, 40], fungus [41–44], bacteria [45–49], yeast [50–52], and viruses [53, 54]. Biological entities offer a nontoxic, clean, and environment-friendly approach to synthesize the NPs with a wide range of size, physicochemical properties, shapes, and compositions [55].

Copper nanoparticles were synthesized and stabilized in the literature by using different plants such as *Euphorbia esula* [56], *Punica granatum* [57], *Ocimum sanctum* [58], *Ginkgo biloba* [59], *Calotropis procera* [60], *Lawsonia inermis* [61], *Citrus medicalinn* [62], *Camellia sinensis* [63], *Datura innoxia* [64], *Syzygium aromaticum* [65], *Sesamum indicum* [66], *Citrus limon*, *Turmeric curcumin* [67], *Gloriosa superba* L. [68], *Ficus carica* [69], *Aegle marmelos* [70], *Caesalpinia pulcherrima* [71], *Cassia fistula* [72], *Leucas aspera*, *Leucas chinensis* [73], *Delonix elata* [74], *Aloe barbadensis* Miller [75], *Thymus vulgaris* [76], *Phyllanthus emblica* [77], *Magnolia kobus* [78], *Eucalyptus* [79], *Artabotrys odoratissimus* [80], *Capparis zeylanica* [81], *Vitis vinifera* [82], *Hibiscus rosa-sinensis* [83], *Zingiber officinale* [84], *Datura metel* [85], *Zea mays* [86], *Urtica*, *Matricaria chamomilla*, *Glycyrrhiza glabra*, *Schisandra chinensis*, *Inula helenium*, *Cinnamomum* [87], *Dodonaea viscosa* [88], *Cassia auriculata* [89], *Azadirachta indica*, *Lantana camera*, *Tridax procumbens* [90], *Allium sativum* [91], *Asparagus adscendens*, *Bacopa monnieri*, *Ocimum bacilicum*, *Withania somnifera* [92], *Smithia sensitiva*, *Colocasia esculenta* [93], *Nerium oleander* [94], and *Psidium guajava* [95]; by using different algae/fungi such as *Phaeophyceae* [96], *Stereum hirsutum* [97], and *Hypocrea lixii* [98]; and by using some microorganisms such as *Pseudomonas fluorescens* [99] and *Enterococcus faecalis* [100] cultures.

## Biosynthesis of Copper Nanoparticles

### Parts of Plant Used for Extract

Different parts of plants are used for the preparation of plant extracts such as leaves, seeds, barks, fruits, peel, coir, roots, and gum. Leaves and roots are used in two ways. Firstly, fresh leaves and roots are used for the preparation of plant extracts, and secondly, dry leaves and roots in powder form are used.

### Procedure for the Synthesis of CuNPs

For the synthesis of CuNPs, plant extract was prepared by using different parts of different plants. For synthesis of the extract part of the plant of interest, leaves are collected and washed with tap water and then with distilled water to remove dust particles. The washed leaves are used further in two ways. First, these leaves are sun dried for 1–2 h to remove the residual moisture. Known weights of these sun-dried leaves are divided into small parts and soaked in deionized water or ethanol solution. This mixture is stirred for 24 h at room temperature by using a magnetic stirrer and then filtered for further use. Second, these leaves are sun dried for 4–7 days or dried in an oven at 50 °C for 1 day and powdered using a domestic blender. Known weight of plant powder is mixed in water or ethanol solution and then stirred and filtered.

For the synthesis of CuNPs, aqueous solution of precursor salts such as copper sulfate, copper chloride, copper acetate, and copper nitrate with different concentrations is mixed with plant extract. Aqueous solution of sodium hydroxide is also prepared and added to the reaction mixture to control the pH medium. The reaction mixture is strongly shaken for different time intervals in an electric shaker and heated in an oven at different time intervals and at different temperatures. The formation of CuNPs can also take place at room temperature and is confirmed by changing the color of the reaction mixture. At the end, nanoparticles were centrifuged and dried at different temperatures. Reaction optimizations take place by changing the pH of the mixture, concentration of precursor salt, heating time, and temperature of reaction mixture. In the literature, different plants have been used for the formation of copper nanoparticles by using different precursor salts with different reaction conditions as shown in Table 1. From the table, it can be seen that the different reaction conditions affect the shape and size of copper nanoparticles.

**Table 1:** Data for synthesis of copper nanoparticles under different reaction conditions

Plants	Part of plant	Active compounds in plant	Precursor salt	Concentration of salt	Reaction conditions	Characterization	Size	Shape	References
*Euphorbia esula*	Leaves	Flavonoids and phenolic acids	Copper chloride	5 mM	Temp 120 °C, pH 9, time 20 min	UV, FTIR, XRD, TEM	20–110 nm	Spherical	[56]
*Punica granatum*	Peels	–	Copper sulfate	50 mM	Temp 80 °C for 10 min and 40 °C for 4 h	UV, FTIR, PSA, TEM	15–20 nm	Spherical	[57]
*Ocimum sanctum*	Leaves	Terpenoids, alcohols, ketones, esters, aldehydes, and carboxylic acids	Copper sulfate	1 mM	Room temp	UV, FTIR, PSA, TEM, MZS	25 nm	Rod, cylindrical, elliptical	[58]
	Leaves		Copper sulfate	1 mM	Room temp	UV, FTIR, EDX, SEM	150–200 nm	Spherical	[115]
*Ginkgo biloba*	Leaves	Polyphenols, quercetin	Copper chloride	5 mM	Temp 80 °C, pH 9, time 30 min	UV, FTIR, EDS, TEM	15–20 nm	Spherical	[59]
*Calotropis procera*	Latex	Cysteine proteases	Copper acetate	3 mM	Room temp	UV, FTIR, XRD, TEM, EDAX	15 ± 1.7 nm	Spherical	[60]
*Lawsonia inermis*	Leaves	–	Copper sulfate	10 mM	Temp 100 °C, pH 11, time 30 min	UV, FTIR, HRTEM, SEM, DMOM		–	[61]
*Citrus medicalinn*	Fruit juice	Ascorbic acid, saponins, and flavonoids	Copper sulfate	100 mM	Temp 60–100 °C	UV, FTIR, NTA, XRD	33 nm	–	[62]
*Camellia sinensis*	Leaves	Flavonoids, phenolic acids, terpenoids, and polysaccharides	Copper chloride	1 mM	Temp 100 °C, time 3 h	UV, FTIR, EDX, TEM, SEM	15–25 nm	Spherical	[63]
	Leaves	–	Copper chloride	10 mM	Temp 90 °C	FTIR, EDX, TEM, SEM, XRD, NTA	10–40 nm	Spherical	[104]
*Datura innoxia*	Leaves	–	Copper sulfate	1 mM	–	UV, FTIR, EDX, FESEM	90–200 nm	Spherical	[64]
*Syzygium aromaticum*	Flowers	Eugenol	Copper sulfate	1 mM	Room temp, pH 3.43	UV, FTIR, XRD, TEM, SEM	5–40 nm	–	[65]
*Sesamum indicum*	Seeds	–	Copper sulfate	10 mM	–	UV	–	–	[66]
*Citrus limon* and *Turmeric curcumin*	Fruit	Curcuminanilineazomethine	Copper chloride	1 mM	–	UV, FTIR, XRD, HRTEM, SEM	60–100 nm	Spherical	[67]
*Gloriosa superba* L.	Leaves	–	Copper sulfate	1 mM	Room temp	UV, FTIR	–	–	[68]
*Gossypium*	Gum	Hydroxyl, acetyl, carbonyl, and carboxylic groups	Copper nitrate	40 mM	Room temp, pH 12	TEM, SAXS, UV, XRD	19 nm	Spherical	[116]
*Ficus carica*	Leaves	–	Copper chloride	10 mM	Temp 25 °C, pH 8, time 30 min	UV, SEM, XRD	50–120 nm	–	[69]
*Aegle marmelos*	Leaves	Polyphenols, alkenoids, phenylpropanoid, and terpenoids	Copper chloride	1 mM	–	UV, FTIR, XRD	48 nm	Spherical	[70]
*Caesalpinia pulcherrima*	Flowers	–	Copper nitrate	1 mM	–	UV, FTIR, XRD, SEM, EDAX	18–20 nm	Spherical	[71]
*Cassia fistula*	Flowers	–	Copper sulfate	1 mM	Room temp	UV, FTIR, XRD, SEM	20	–	[72]
*Leucas aspera*	Leaves	–	Copper sulfate	1 mM	–	UV	–	–	[73]
*Leucas chinensis*	Leaves	–	Copper sulfate	1 mM	–	XRD, FESEM, EDX	60.23 nm	–	[117]
*Delonix elata*	Flowers	–	Copper sulfate	1 mM	–	UV, FTIR, XRD, SEM	20	–	[74]
*Aloe barbadensis* Miller	Flowers	–	Copper acetate	5 mM	Temp 50 °C, time 30 min	UV, FTIR, FESEM	40 nm	Spherical	[75]
*Thymus vulgaris*	Leaves	–	Copper sulfate	0.2 M	Temp 80 °C, time 4 h	BET, TEM, SAED, FTIR, XRD, XRF, FESEM, EDS	–	–	[76]
*Phyllanthus emblica*	Fruit	Tannin, saponin, flavonoid, alkaloid, quinone, anthraquinone, anthocyanosides, phenols	Copper sulfate	20 mM	Temp 60–80 °C, pH 10	UV, FTIR, XRD, SEM, EDAX	15–30 nm	Flakes	[77]
*Magnolia kobus*	Leaves		Copper sulfate	1 mM	Temp 25–95 °C	ICP, EDS, XPS, SEM, HRTEM	40–100 nm	Spherical	[78]
*Eucalyptus*	Leaves	Flavonoids and phenolic acids	Copper sulfate	1 mM	–	UV, FTIR, XRD	38.62 nm	–	[79]
*Artabotrys odoratissimus*	Leaves	–	Copper sulfate	1 mM	Temp 95 °C	PSA	35 nm	–	[80]
*Capparis zeylanica*	Leaves	–	Copper sulfate		–	UV, FTIR, SEM, EDX, XRD, TEM	50–100 nm	Cubical	[81]
*Vitis vinifera*	Leaves	–	Copper acetate	1%	–	UV, FTIR, XRD	3–6 nm	–	[82]
*Hibiscus rosa-sinensis*	Leaves	Polyphenols, flavonoids, proteins, lignins, xanthones	Copper nitrate	50 mM	–	UV, FTIR, TEM	–	–	[83]
*Zingiber officinale*	–	–	–	–	–	FTIR, XRD, EDX, TEM, SAED	10.13 nm	Cubical	[84]
*Datura metel*	Leaves	Alkaloids, terpenoids, and phenolic groups	–	–	Time 10 min	UV, PSA, TEM, EDX, FTIR	–	–	[85]
*Zea mays*	Leaves	–	Copper sulfate	10 mM	Room temp, time 1 h	UV, XRD, EDAX, FTIR	40 nm	Mixed	[86]
*Urtica*	Leaves	Flavonoids, quercetin, rutin, morin	Copper sulfate	–	Temp 70 °C	UV, SEM, XRD	6.5 nm	–	[87]
*Matricaria chamomilla*	Leaves	Flavonoids	Copper sulfate	–	Temp 70 °C	UV, SEM, XRD	58.77 nm	–	[87]
*Glycyrrhiza glabra*	Leaves	Flavonoids	Copper sulfate	–	Temp 70 °C	UV, SEM, XRD	28.21 nm	–	[87]
*Schisandra chinensis*	Leaves	Quercetin, rutin, morin	Copper sulfate	–	Temp 70 °C	UV, SEM, XRD	32 nm	–	[87]
*Inula helenium*	Leaves	Flavonoids	Copper sulfate	–	Temp 70 °C	UV, SEM, XRD	32.41 nm	–	[87]
*Cinnamomum*	Leaves	Flavonoids	Copper sulfate	–	Temp 70 °C	UV, SEM, XRD	48.8 nm	–	[87]
*Dodonaea viscosa*	Leaves	Santin, penduletin, alizarin, pinocembrin, tannins, saponins	Copper chloride	1 mM	Temp 50 °C, pH 10	UV, XRD, AFM, HRTEM, SAED	30–40 nm	Spherical	[88]
*Cassia auriculata*	Leaves	–	Copper sulfate	1 mM	–	FESEM, XRD, FTIR	38–43 nm	Spherical	[89]
*Azadirachta indica*	Leaves	–	Fehling solution	–	–	UV	–	–	[90]
*Lantana camera*	Leaves	–	Fehling solution	–	–	UV	–	–	[90]
*Tridax procumbens*	Leaves	–	Fehling solution	–	–	UV	–	–	[90]
*Allium sativum*		–	Copper sulfate	10 mM	–	UV, FTIR, SEM, XRD, TEM	100 nm	Spherical	[91]
*Asparagus adscendens*	Leaves	–	copper sulfate	1 mM	–	UV, FTIR, TEM, SAED	10–15 nm	Spherical	[92]
*Bacopa monnieri*	Leaves	–	copper sulfate	1 mM	–	UV, FTIR, TEM, SAED	50–60 nm	Spherical	[92]
*Ocimum bacilicum*	Leaves	–	copper sulfate	1 mM	–	UV, FTIR, TEM, SAED	40–60 nm	Spherical	[92]
*Withania somnifera*	Leaves	–	copper sulfate	1 mM	–	UV, FTIR, TEM, SAED	50–60 nm	Mixed	[92]
*Smithia sensitiva*	Leaves	Tannin, saponin, flavonoid, anthraquinone glycoside, steroids	Copper sulfate	1 mM	–	UV, FTIR, SEM, NTA	136 nm	–	[93]
	Leaves	Tannin, saponin, flavonoid, anthraquinone glycoside, steroids	Copper acetate	1%	–	UV, FTIR, SEM, NTA	50 nm	–	[93]
*Colocasia esculenta*	Leaves	Tannin, flavonoid, alkaloid, cardiac glycoside, terpenoids, phenols	Copper sulfate	1 mM	–	UV, FTIR, SEM, NTA	57 nm	–	[93]
	Leaves	Tannin, flavonoid, alkaloid, cardiac glycoside, terpenoids, phenols	Copper acetate	1%	–	UV, FTIR, SEM, NTA	44 nm	–	[93]
*Nerium oleander*	Leaves		Copper sulfate	1 mM	–	UV, FTIR	–	–	[94]
*Psidium guajava*	Fruit	Flavonoid, alkaloid, steroids, glycoside, terpenoids, phenols	Copper sulfate	20 mM	Room temp, pH 10	UV, FTIR, XRD, EDAX, TEM, SEM	15–30 nm	Flakes	[95]

### Effect of Reaction Parameters on Properties of NPs

The concentration of plant extract plays a main role in reducing and stabilizing the CuNPs. It has been reported that by increasing the concentration of plant extract, the number of particles increased [88]. By increasing the concentration of plant extract, the concentration of phytochemicals increased and the reduction of copper salt also increased. Due to the fast reduction of the metal salt, the size of the nanoparticles also decreased [101].

The size and structure of CuNPs are highly affected by the copper salt. The morphology of nanoparticles changes when the salt (e.g., copper chloride, copper acetate, copper nitrate, or copper sulfate) is used in the presence of sodium hydroxide. It was reported that the shape was triangular and tetrahedron in the case of copper chloride, rod-shaped in the case of copper acetate, and spherical in the case of copper sulfate [102]. By increasing the concentration of the precursor salt, the size of the CuNPs also increased.

**Fig. 1:**
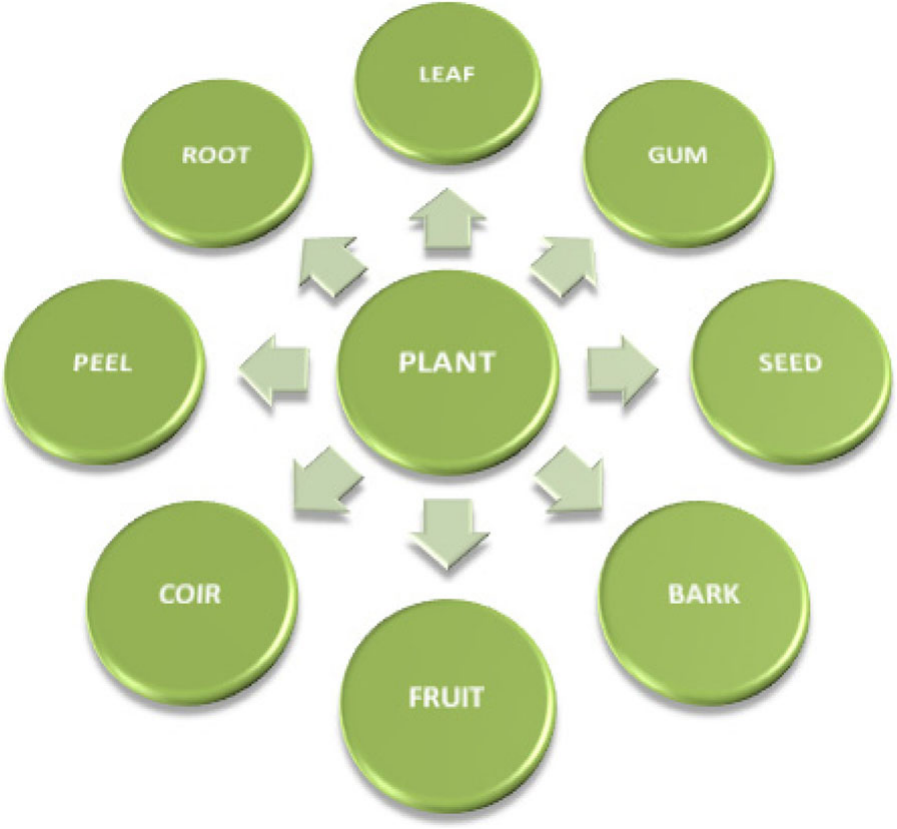
Parts of the plant used for the preparation of plant extract

The synthesis of CuNPs gives best results by varying the pH of the reaction medium within the preferred range. The size of nanoparticles was controlled by changing the pH value of the reaction mixture. At higher pH, smaller-sized nanoparticles were obtained compared to those obtained at low pH value. This difference can be attributed to the difference in reduction rate of the metal salts by plant extract. The inverse relation between the value of pH and the size of nanoparticle showed that an increase in pH value enables us to obtain small-sized spherical nanoparticles while a decrease in pH value gives large-sized (rod-shaped and triangular) nanoparticles. The effect on absorption spectra of different values of pH (4, 6, 8, 10, and 12) is represented in Fig. 1 [36]. It was reported that the addition of plant extract to CuCl_2_ did not lead to the formation of CuNPs but, instead, the CuNPs were obtained by changing the pH of the reaction mixture to basic medium. The same behavior was observed by Wu and Chen, and it was concluded that pH plays an important role in the synthesis of CuNPs [103].

## Mechanism for Phytosynthesis of Copper Nanoparticles

### Phytochemical Screening: a Qualitative Analysis

Phytochemical screening analysis is a chemical analysis carried out for the detection of phytochemicals in different plants. Fresh plant extract with chemicals or chemical reagents is used for this analysis [77] as shown in Table 2.

**Table 2:** Phytochemical screening analysis

Test for phytochemicals	Amount of plant extract	Chemicals used	End point for confirmation of phytochemical
Carbohydrate	2 mL	Few drops of concentrated sulfuric acid and 1 mL of Molisch’s reagent	Reddish or purple color
Tannins	2 mL	4 mL of 5% ferric chloride	Greenish black or dark blue color
Saponins	2 mL	2 mL of distilled water and shake for 15 min	Layer of foam on surface
Flavonoids	2 mL	1 mL of 2 N sodium hydroxide	Yellow color
Alkaloids	2 mL	Few drops of Mayer’s reagent and 2 mL of concentrated HCl	White precipitate or green color
Anthraquinone	1 mL	Few drops of 10% ammonia solution	Pink color precipitates
Anthocyanosides	1 mL of filtrate	5 mL HCl	Pale pink color

### Phytochemicals for Reduction of Metal and Stabilizing the NPs

**Fig. 2:**
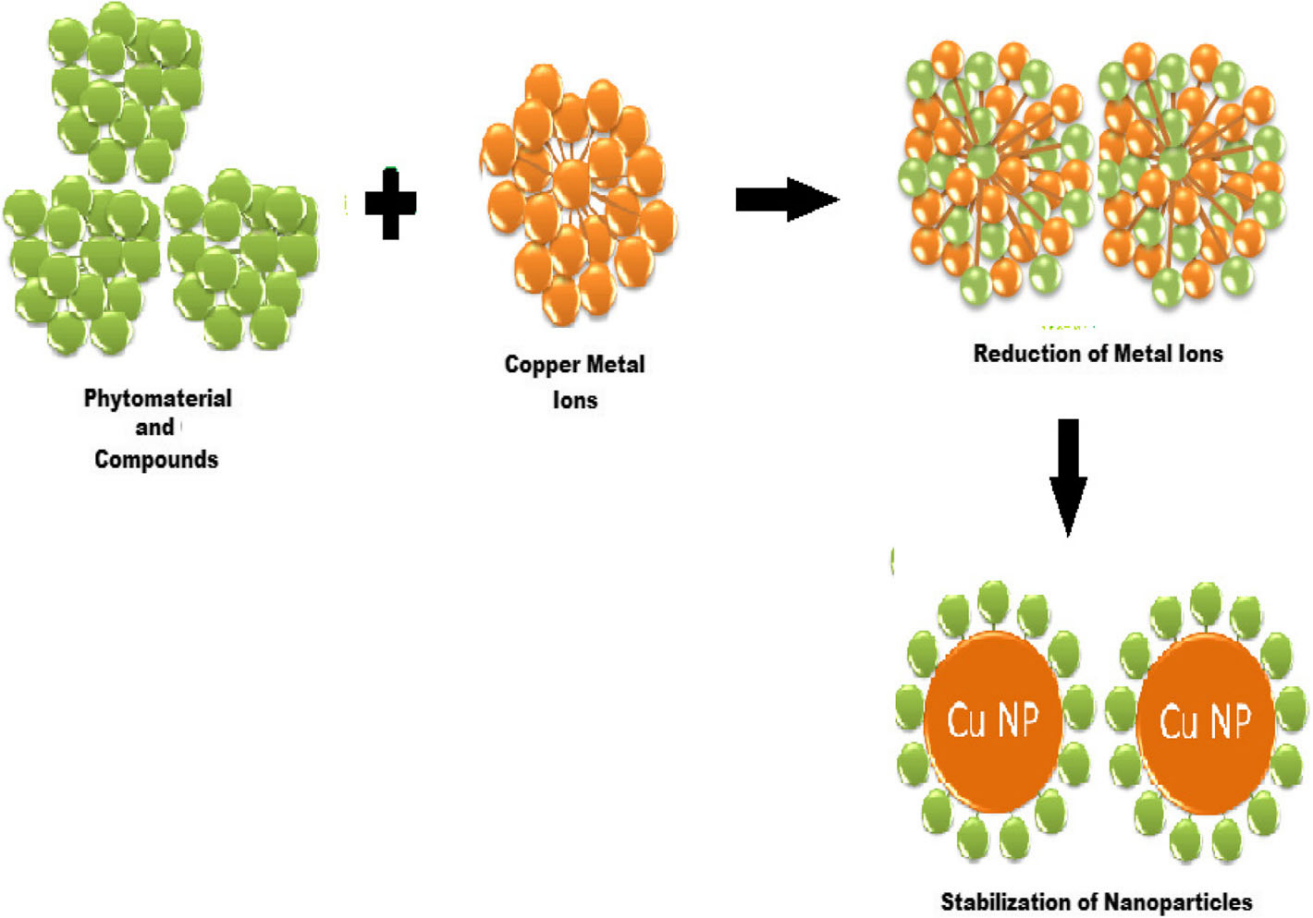
A protocol for reducing the metal ions and then stabilizing the metal’s nuclei

Green synthesis of CuNPs by the use of phytochemicals offers more flexible control over the shape and size of the NPs (i.e., by changing reaction temperature, concentration of plant extract, metal salt concentration, reaction time, and pH of reaction mixture). Color change of the reaction medium indicates reduction of the metal ion and formation of NPs. The green reduction of the copper salts starts instantly, and the formation of copper nanoparticles is indicated by the color change of the reaction mixture. Phytochemicals have a main role in first reducing the metal ions and then stabilizing the metal’s nuclei in the form of nanoparticles as shown in Fig. 2. The interaction of phytochemicals with metal ions and the concentration of these phytochemicals control the shape and size of CuNPs.

**Fig. 3:**
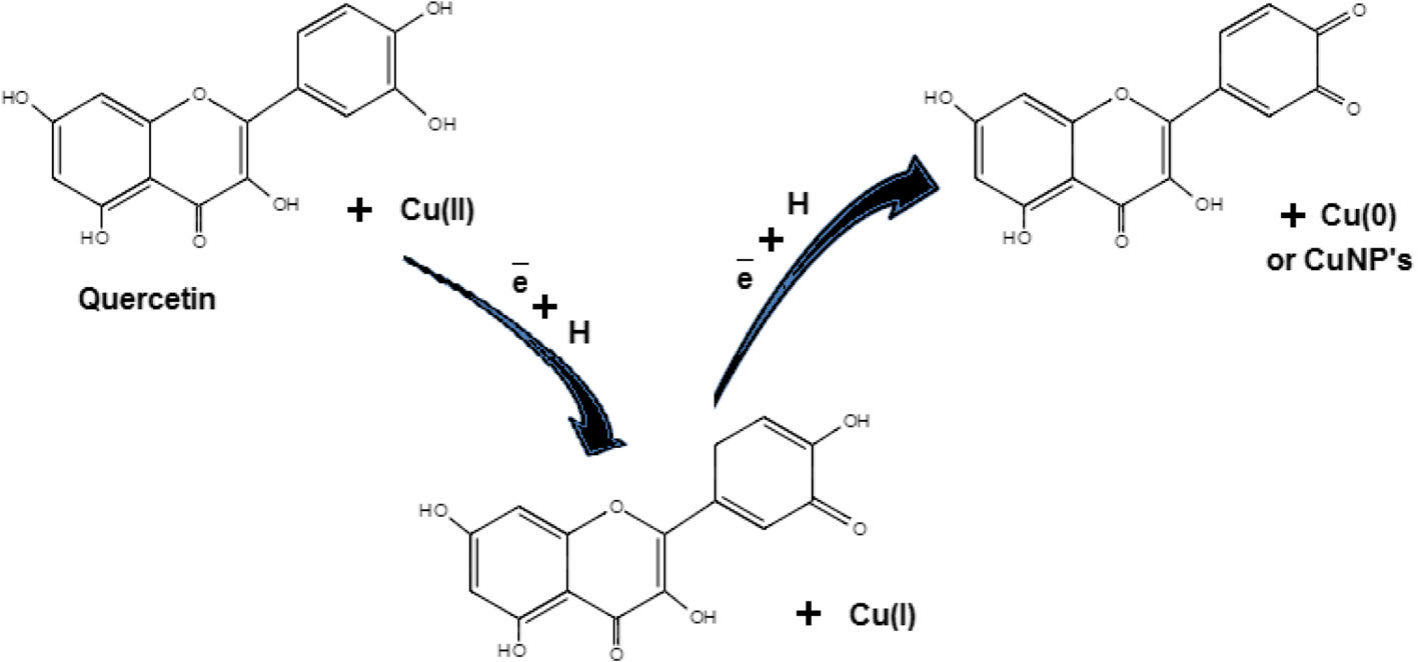
Reduction of copper ions by quercetin

Flavonoids contain polyphenolic compounds, e.g., quercetin, catechins, flavanones, isoflavones, santin, penduletin, alizarin, pinocembrin, anthocyanins, flavones, tannins, and saponins, which are present in different plants such as *Ginkgo biloba* [59], *Citrus medicalinn* [62], *Phyllanthus emblica* [77], *Hibiscus rosa-sinensis* [83], and *Dodonaea viscosa* [93]. These compounds play a main role in reducing and chelating the metal. Various functional groups present in the flavonoids are responsible for the reduction of the copper ion. It has been assumed that a reactive hydrogen atom in the flavonoids may be released during the tautomeric alterations of the enol form to the keto form which can reduce copper ions to form copper nuclei or CuNPs. For example, it is assumed that in the case of *Ginkgo biloba* plant extracts, it is the transformation of quercetin (flavonoid) which plays a main role in the reduction of copper metal ions into copper nuclei or CuNPs due to the change of enol form to keto form as shown in Fig. 3.

**Fig. 4:**
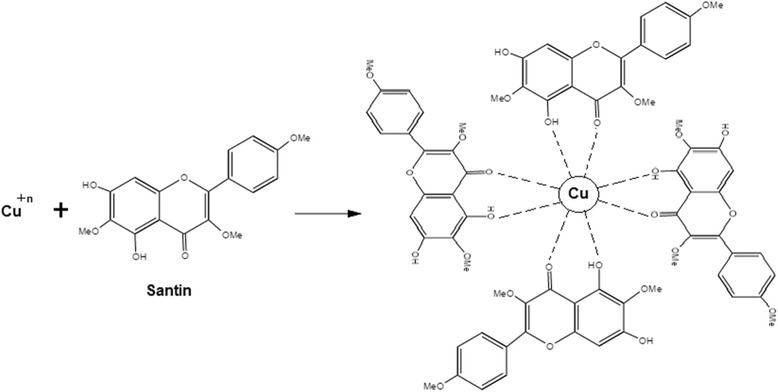
Stabilization of copper nanoparticles by santin

During the synthesis process of CuNPs, metal ions with monovalent or divalent oxidation states are converted into zero-oxidation copper nuclei and these nuclei are merged to obtain different shapes. During the nucleation, nuclei aggregate to form different shapes such as wires, spheres, cubes, rods, triangles, pentagons, and hexagons. Some flavonoids have an ability to chelate the CuNPs with their *π* electrons and carbonyl groups. Quercetin and santin are flavonoids with strong chelating activity due to the presence of two functional groups involving the hydroxyls and carbonyls. These groups chelate with copper nanoparticles by following the previous mechanism and also explain the ability of adsorption of santin (flavonoid) on the surface of CuNPs as shown in Fig. 4.

It was assumed that the protein molecules (superoxide dismutase, catalase, glutathione) in different plants such as *Hibiscus rosa-sinensis* [83] and *Camellia sinensis* [104] display a high reducing activity for the formation of nanoparticles from metal ions but their chelating activity is not excessive. Sugars such as monosaccharides (glucose), disaccharides (maltose and lactose), and polysaccharides in *Camellia sinensis* plant [63] can act as reducing agents or antioxidants and have a series of tautomeric transformations from ketone to aldehyde.

**Fig. 5:**
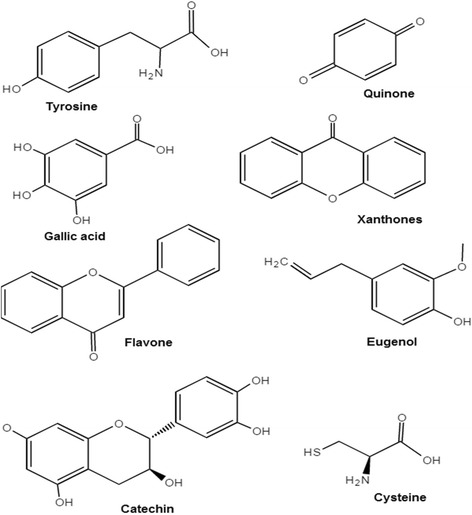
Phytochemicals with their structures

Other phytochemicals such as polyphenols (e.g., ellagic acid and gallic acid) which are present in *Hibiscus rosa-sinensis* [40], phenylpropanoids (phenylalanine, tyrosine) in *Aegle marmelos* [70], terpenoids in *Ocimum sanctum* and *Asparagus adscendens* [58, 92], cysteine proteases in *Calotropis procera* [60], curcuminanilineazomethine in *Turmeric curcumin* [67], ascorbic acid in *Citrus medicalinn* [62], eugenol in *Syzygium aromaticum* [65], and alkaloids in *Aegle marmelos* [70] play the same role of reducing the copper ions and stabilizing the copper nanoparticles. Carbohydrates, anthraquinone, quinone, and anthocyanoside in *Phyllanthus emblica* [77]; lignins and xanthones in *Hibiscus rosa-sinensis* [83]; and cardiac glycoside, triterponoid, carotenoid glycoside, and anthraquinone glycoside in *Colocasia esculenta* plant [93] are also phytochemicals which are present in extracts of different plants and act as reducing and stabilizing agents. Examples of certain phytochemicals with structures are shown in Fig. 5.

## Characterization Techniques

For characterization of synthesized nanoparticles, different techniques were used such as ultraviolet-visible spectroscopy (UV-vis), transmission electron microscopy (TEM), small-angle X-ray scattering (SAXS), Fourier transform infrared spectroscopy (FTIR), X-ray fluorescence spectroscopy (XRF), X-ray diffraction (XRD), X-ray photoelectron spectroscopy (XPS), scanning electron microscopy (SEM), field emission scanning electron microscopy (FESEM), particle size analysis (PSA), Malvern Zetasizer (MZS), energy-dispersive X-ray spectroscopy (EDX/EDS), nanoparticle tracking analysis (NTA), X-ray reflectometry (XRR), Brunauer-Emmett-Teller analysis (BET), selected area electron diffraction (SAED), and atomic force microscopy (AFM) (Table 3).

**Table 3:** Characterization techniques and limitations

Technique	Main role	Limitations	Sensitivity	Ref.
Ultraviolet-visible spectroscopy (UV-vis)	Concentration and shape of NPs can be measured	Only for liquid samples	UV-visible regions 200–800 nm	[22]
Fourier transform infrared spectroscopy (FTIR)	Nature of bonds and functional groups can be determined	Structure and size of NPs cannot be measured	20 Å–1 μm	[22]
X-ray diffraction (XRD)	Size and crystallinity of nanoparticles can be measured	Composition of NPs and plasmon cannot be found	1 nm	[36]
Scanning electron microscopy (SEM)	Shape and size of nanostructures can be determined	Samples must be solid and cannot detect elements with atomic number < 11	< 1 nm	[115]
Field emission scanning electron microscopy (FESEM)	All structural and morphological investigations are carried out by this technique	Does not give a concentration of NPs	< 1 nm	[117]
Transmission electron microscopy (TEM)	Shape and size of nanostructures can be determined	Particles with size < 1.5 nm cannot be determined	< 1.5 nm	[92]
Particle size analysis (PSA)	Measured the distribution of size in the sample of solid or liquid particulate materials	–	1 nm–1 μm	[57, 58]
Malvern Zetasizer (MZS)	Measured the size of NPs, zeta potential, and protein mobility	In nanorange	–	[58]
Energy-dispersive X-ray spectroscopy (EDX/EDS)	Composition of NPs can be analyzed	Particles with size < 2 nm cannot be analyzed	< 2 nm	[59, 60]
Nanoparticle tracking analysis (NTA)	Visualize and measure particle size, concentration, and fluorescent properties of a nanoparticle	–	30–10 nm	[62]
Small-angle X-ray scattering (SAXS)	Size and shape conformation	Lower resolution range	50–10 Å	[116]
X-ray reflectometry (XRR)	Determination of thickness, density, and roughness	Layer thickness 0.1–1000 nm	–	[116]
X-ray fluorescence spectroscopy (XRF)	Chemical composition and concentration can be measured	Limited in their ability to measure precisely and accurately	–	[76]
X-ray photoelectron spectroscopy (XPS)	Elemental composition of nanoparticles can be analyzed	Decomposition of samples occurred	3–92 nm	[78]
Brunauer-Emmett-Teller analysis (BET)	Specific surface area is measured		0.35–2 nm	[76]
Selected area electron diffraction (SAED)	Technique that can be performed inside a TEM	Cannot be recommended for quantitative identification techniques	–	[76]
Atomic force microscopy (AFM)	Particle size and characterization	For gas and liquid samples	1 nm–8 μm	[88]

## Applications of Copper Nanoparticles

Due to their outstanding chemical and physical properties, large surface-to-volume ratio, constantly renewable surface, low cost, and nontoxic preparation, CuNPs have been of great interest for applications in different fields. Copper nanoparticles show catalytic activity, antibacterial activity, cytotoxicity or anticancer activity, antioxidant activity, and antifungal activity in different applications. In catalytic activity, copper nanoparticles are used for the Huisgen [3 + 2] cycloaddition of alkynes and azides in many solvents under ligand-free conditions [59], 1-methyl-3-phenoxy benzene, 3,3-oxybis(methylbenzene) [94], synthesis of 1-substituted 1*H*-1,2,3,4-tetrazole [76], adsorption of nitrogen dioxide, and adsorption of sulfur dioxide [66]. In most of the transition metals catalyzed, Ullmann coupling-reaction ligands, such as phosphines, are reported in the literature and most ligands are expensive, difficult to prepare, and moisture sensitive. For this work, synthesized copper nanoparticles are used for ligand-free Ullmann coupling of diphenyl ether. Different dyes and toxic organic compounds and pesticides present in industrial waste are very harmful for the environment and living organisms. Copper nanoparticles are used for degradation of different dyes such as methylene blue [73], degradation of atrazine [86], and reduction of 4-nitrophenol [76].

Among the antimicrobial agents, copper compounds have been commonly used in agriculture as herbicides [105], algaecides [106], fungicides [107], and pesticides as well as in animal husbandry as a disinfectant [108] (shown in Table 4). The biogenic copper nanoparticles showed powerful antibacterial activity against gram-positive and gram-negative pathogens such as *Pseudomonas aeruginosa* (MTCC 424), *Micrococcus luteus* (MTCC 1809), *Enterobacter aerogenes* (MTCC 2832) [57], *Salmonella enterica* (MTCC 1253), *Rhizoctonia solani*, *Xanthomonas axonopodis* pv. *citri*, *Xanthomonas axonopodis* pv. *punicea* [58], *Escherichia coli* (ATCC 14948) [62], *Staphylococcus aureus* (ATCC 25923), *Bacillus subtilis* (ATCC 6633), *Pediococcus acidilactici* [69], and *Klebsiella pneumoniae* (MTCC 4030). In antifungal activity, copper nanoparticles are used against *Alterneria carthami*, *Colletotrichum gloeosporioides*, *Colletotrichum lindemuthianum*, *Drechslera sorghicola*, *Fusarium oxysporum* f.sp. *carthami*, *Rhizopus stolonifer*, *Fusarium oxysporum* f.sp. *ciceris*, *Macrophomina phaseolina*, *Fusarium oxysporum* f.sp. *udum*, *Rhizoctonia bataticola* [58], *Candida albicans*, *Curvularia*, *Aspergillus niger*, and *Trichophyton simii* [67]. In cytotoxicity, copper nanoparticles are used for a study on HeLa, A549, MCF7, MOLT4, and BHK21 cell lines (cancer tumors) [60, 104].

**Table 4:** Catalytic, antibacterial, cytotoxicity or anticancer, antioxidant, and antifungal activities of copper nanoparticles

Biological entity	Activity	In/against	Concentration of NPs	References
*Euphorbia esula*	Catalytic	Reduction of 4-nitrophenol	25 μL	[56]
	Catalytic	Ligand-free Ullmann coupling of diphenyl ether, 1-methyl-3-phenoxy benzene, and 3,3-oxybis(methylbenzene)	1 mL	[56]
*Punica granatum*	Antibacterial	*Enterobacter aerogenes*, *Micrococcus luteus*, *Salmonella enterica*, and *Pseudomonas aeruginosa*	100 μg/L	[57]
*Ocimum sanctum*	Antibacterial	*Rhizoctonia solani*, *Xanthomonas axonopodis* pv. *citri*, *Xanthomonas axonopodis* pv. *punicea*	–	[58]
	Antifungal	*Alterneria carthami*, *Colletotrichum gloeosporioides*, *Colletotrichum lindemuthianum*, *Drechslera sorghicola*, *Fusarium oxysporum* f.sp. *carthami*, *Rhizopus stolonifer*, *Fusarium oxysporum* f.sp. *ciceris*, *Macrophomina phaseolina*, *Fusarium oxysporum* f.sp. *udum*, and *Rhizoctonia bataticola*	–	[58]
*Ginkgo biloba*	Catalytic	Huisgen [3 + 2] cycloaddition of azides and alkynes	10 mol%	[59]
*Calotropis procera*	Cytotoxicity	Study on HeLa, A549, and BHK21 cell lines (cancer tumors)	120 μM	[60]
*Citrus medicalinn*	Antibacterial	*Propionibacterium acne*s (MTCC 1951), *Salmonella typhi* (ATCC 51812), *K. pneumoniae* (MTCC 4030), *P. aeruginosa*, and *Escherichia coli*	20 μL	[62]
	Antifungal	*Fusarium culmorum* (MTCC 349) and *Fusarium oxysporum* (MTCC 1755)	20 μL	[62]
*Camellia sinensis*	Antibacterial	*Pseudomonas aeruginosa*, *Escherichia coli*, *Staphylococcus aureus*, and *Bacillus subtilis*	2, 4, 6, and 8 μg/L	[63]
	Anticancer	HT-29, MCF7, and MOLT4 cell lines	80 μg/mL	[104]
*Datura innoxia*	Antibacterial	*Xanthomonas oryzae* pv. *oryzae*		[64]
*Sesamum indicum*	Catalytic	Adsorption of nitrogen dioxide and sulfur dioxide	0.01–0.06 g	[66]
*Citrus limon* and *Turmeric curcumin*	Antibacterial	*Pseudomonas aeruginosa*, *Escherichia coli*, *Staphylococcus aureus*, and *Bacillus subtilis*	–	[67]
	Antifungal	*Candida albicans*, *Curvularia*, *Aspergillus niger*, *Trichophyton simii*	–	[67]
*Ficus carica*	Antibacterial	*Pediococcus acidilactici*	10 μg/mL	[69]
*Leucas aspera*	Catalytic	Degradation of methylene blue	1 mL	[73]
*Thymus vulgaris*	Catalytic	Reduction of 4-nitrophenol and synthesis of 1-substituted 1*H*-1,2,3,4-tetrazole	50 g and 15 mg, respectively	[76]
*Phyllanthus emblica*	Antibacterial	*Staphylococcus aureus* and *Escherichia coli*	–	[77]
*Magnolia kobus*	Antibacterial	*Escherichia coli* (ATCC 25922)	–	[78]
*Capparis zeylanica*	Antibacterial	Gram-positive and gram-negative pathogens	–	[81]
*Vitis vinifera*	Antibacterial	*Bacillus subtilis* and *Escherichia coli* (ATCC 25922)	–	[82]
*Hibiscus rosa-sinensis*	Antibacterial	*Bacillus subtilis* and *Escherichia coli* (ATCC 25922)	–	[83]
	Antioxidant	Hydrogen peroxide scavenging assay was assessed	–	[83]
*Zingiber officinale*	Antibacterial	*Staphylococcus aureus* (ATCC 25923), *Bacillus subtilis*, and *Escherichia coli*	–	[84]
*Zea mays*	Catalytic	Degradation of atrazine	30 mg	[86]
*Dodonaea viscosa*	Antibacterial	*Staphylococcus aureus* (ATCC 25923), *Bacillus subtilis*, *Escherichia coli*, and *K. pneumoniae* (MTCC 4030)	–	[88]
*Azadirachta indica*	Antibacterial	*Escherichia coli*	–	[90]
*Lantana camera*	Antibacterial	*Escherichia coli*	–	[90]
	Antifungal	*Aspergillus niger*	–	[90]
*Tridax procumbens*	Antibacterial	*Escherichia coli*	–	[90]
	Antifungal	*Aspergillus niger*	–	[90]
*Allium sativum*	Antibacterial	*Escherichia coli*, *Bacillus subtilis*	75 and 50 μL, respectively	[91]
*Asparagus adscendens*	Antibacterial	*Staphylococcus aureus*	–	[92]
*Bacopa monnieri*	Antibacterial	*Bacillus subtilis*, *Escherichia coli*, *Pseudomonas aeruginosa*	–	[92]
*Nerium oleander*	Antibacterial	*Escherichia coli*, *Staphylococcus aureus*, *Bacillus subtilis*, *K. pneumoniae*, *Salmonella typhi*	35 μL	[94]
*Psidium guajava*	Antibacterial	*Escherichia coli*, *Staphylococcus aureus*	–	[95]

### Hypothetical Mechanism of Antimicrobial Activity

**Fig. 6:**
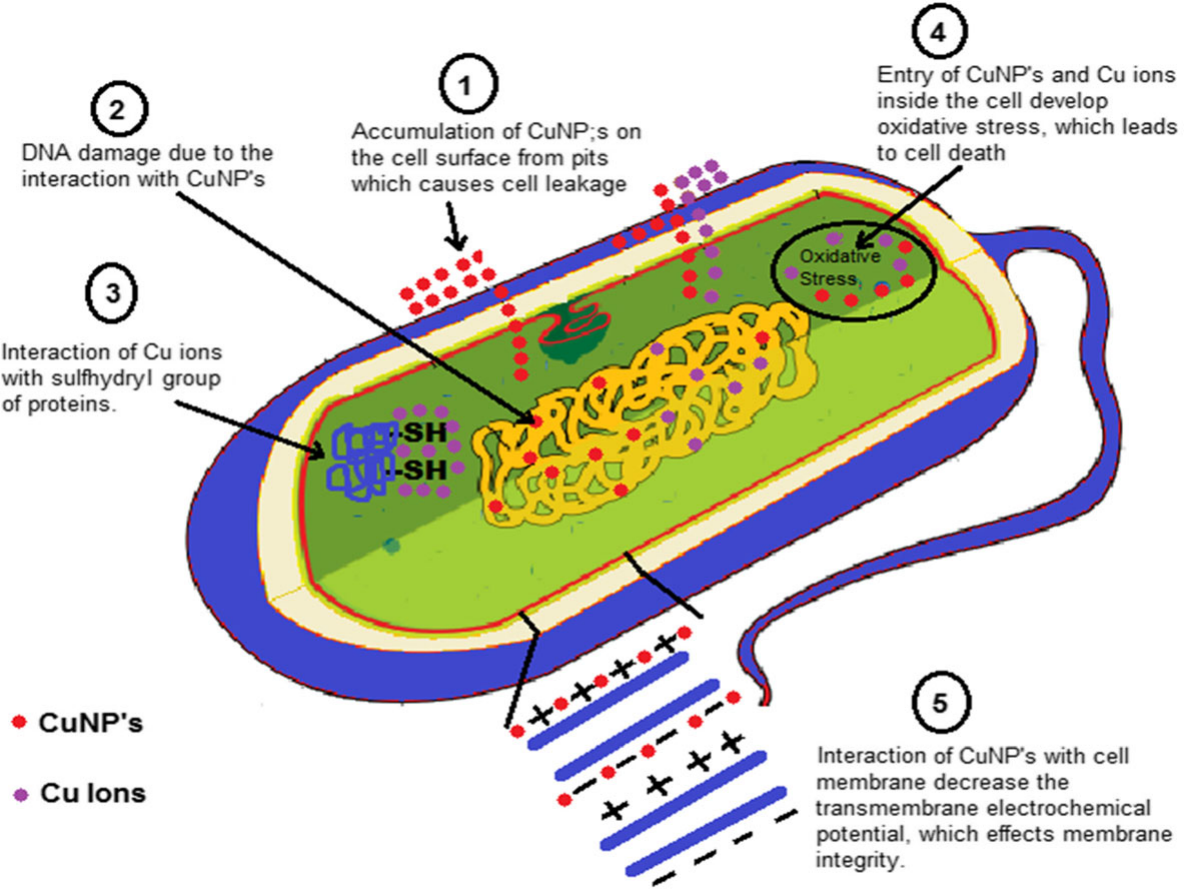
Mechanism for antibacterial activity of copper nanoparticles

It was observed that CuNPs have an excellent antimicrobial activity and only limited reports presented the mechanism of the antibacterial activity of copper nanoparticles in the literature, but these mechanisms were hypothetical. It was observed that bacteria and enzymes/proteins were destroyed due to the interaction of CuNPs with –SH (sulfhydryl) group [109, 110]. It was also reported that the helical structure of DNA molecules become disturbed by the interaction of CuNPs [111]. The interaction of CuNPs with the cell membrane of bacteria decreased the transmembrane electrochemical potential, and due to the decrease in transmembrane electrochemical potential, it affected the membrane integrity [112]. It was assumed that metal NPs release their respective metal ions. Copper nanoparticles and copper ions accumulate on the cell surface of the bacteria and form pits in the membrane, causing leakage of the cellular component from the cell and inside the cell, causing oxidative stress which leads to cell death [112–114]. A hypothetical mechanism of antibacterial activity representing the above possibilities is shown in Fig. 6.

### Catalytic Activity for Reduction of 4-Nitrophenol

4-Nitrophenol (4-NP) which is usually found in agricultural wastewaters and industrial products is hazardous and not environment-friendly. Hydrogenation or reduction of 4-NP, which is converted into 4-aminophenol (4-AP), takes place in the presence of CuNPs. CuNPs can catalyze the reaction to overcome the kinetic barrier by assisting electron transfer from the donor borohydrate ions to the acceptor 4-NP.

**Fig. 7:**
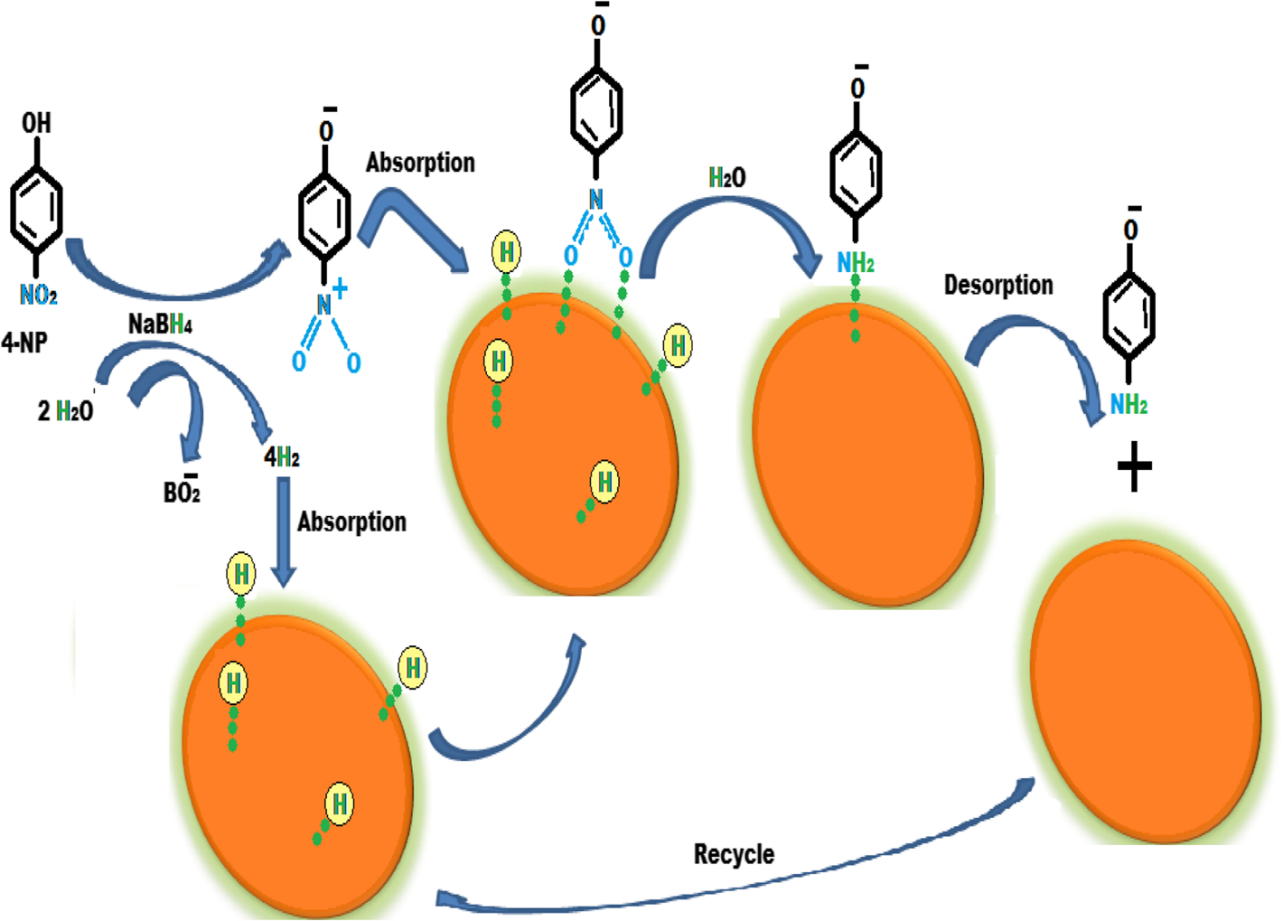
Mechanism for the reduction of 4-nitrophenol

Catalytic activity of the synthesized CuNPs has been studied in the reduction of 4-nitrophenol in aqueous medium at room temperature in the presence of aqueous solution of sodium borohydride [56]. The reduction of 4-NP by using CuNPs is a simple and environment-friendly process. Catalytic efficiency of CuNPs for the reduction of 4-NP was examined by using a UV-vis spectrometer. It was observed that the maximum absorption peak for 4-NP in aqueous medium was at 317 nm and the adsorption peak shifted to 403 nm by adding sodium borohydride due to the formation of 4-nitrophenolate ions. A peak at 403 nm remained unaffected even after 2 days, which indicated that the reduction of 4-NP cannot take place in the absence of a catalyst. After adding the CuNPs, the absorption peak of the solution shifted to 300 nm and the peak at 403 nm completely disappeared which indicated the reduction of 4-NP to 4-AP without any side product. A hypothetical mechanism for the reduction of 4-NP is shown in Fig. 7. In the mechanism, 4-NP and sodium borohydride are present in the solution in the form of ions. The protons of the borohydride ion are adsorbing on the surface of the copper nanoparticles and BO_2_ produced. 4-Nitrophenolate ions also adsorb on the surface of the CuNPs. Due to the adsorption of both protons and 4-nitrophenolate ion, CuNPs overcome the kinetic barrier of reactants and 4-nitrophenolate ion is converted into 4-aminophenolate ion. After conversion, desorption of the 4-aminophenolate ion takes place and it is converted into 4-aminophenol.

## Conclusions

This paper has reviewed and summarized recent information of biological methods used for the synthesis of copper nanoparticles (CuNPs) using different plants. Green synthesis of CuNPs has been proposed as a valuable alternative to physical and chemical methods with low cytotoxicity, economic prospects, environment-friendly, enhanced biocompatibility, feasibility, and high antioxidant activity and high antimicrobial activity of CuNPs. The mechanism of biosynthesis of NPs is still unknown, and more research needs to be focused on the mechanism of formation of nanoparticles and understanding of the role of phytochemicals in the formation of NPs. This review gives data of plants used in the synthesis of copper nanoparticles, synthesis procedure, and the reaction parameters which affect the properties of synthesized CuNPs. A phytochemical screening analysis is a chemical analysis used to identify the phytochemicals such as detection of carbohydrates, tannins, saponins, flavonoids, alkaloids, anthraquinones, and anthocyanosides in different plants. The mechanism of reduction of copper ion by quercetin and stabilization of copper nanoparticles by santin is described in this paper. Characterization techniques used in the literature for copper nanoparticles are UV-vis, FTIR, XRD, SEM, FESEM, TEM, PSA, MZS, EDX, NTA, SAXS, XRR, XRF, XPS, BET, SAED, and AFM. Copper nanoparticles show catalytic activity, antibacterial activity, cytotoxicity or anticancer activity, antioxidant activity, and antifungal activity in different applications. Hypothetical mechanisms of antimicrobial activity and reduction of 4-nitrophenol with diagrams are shown in this paper.

CuNPs with different structural properties and effective biological effects can be fabricated using new green protocols in the coming days. The control over particle size and, in turn, the size-dependent properties of CuNPs will open the new doors of their applications. This study provides an overview of synthesis of CuNP by using plant extract, microbial extract, and naturally occurring biomolecules. Although all these green protocols for CuNP synthesis have their own advantages and limitations, the use of plant extract as a reductant is more beneficial as compared to the use of microbial extract because of the rapid rate of production of nanoparticles with former green reductant.
